# Postoperative pain, pain management, and recovery at home after pediatric tonsil surgery

**DOI:** 10.1007/s00405-020-06367-z

**Published:** 2020-09-26

**Authors:** Fredrik Alm, Stefan Lundeberg, Elisabeth Ericsson

**Affiliations:** 1 Department of Anaesthesia and Intensive Care, School of Health Sciences, Faculty of Medicine and Health, Örebro University, 701 82 Örebro, Sweden; 2 Pain Treatment Service, Department of Physiology and Pharmacology, Karolinska Institute, Astrid Lindgren Children’s Hospital, Karolinska University Hospital, Stockholm, Sweden; 3 School of Health Sciences, Faculty of Medicine and Health, Örebro University, Örebro, Sweden

**Keywords:** Pediatric, Pain, Postoperative recovery, Tonsil surgery, Tonsillectomy, Tonsillotomy, Morbidity, Pain management, Analgesics

## Abstract

**Purpose:**

To explore the severity and duration of postoperative pain, the management of analgesics, and postoperative recovery in children undergoing tonsil surgery.

**Method:**

Participants included 299 children aged 4–17 years undergoing tonsillotomy ± adenoidectomy (TT ± A) or tonsillectomy ± adenoidectomy (TE ± A). Data were collected up to 12 days. The child rated pain on the Face Pain Scale-Revised (FPS-R) and recovery using the Postoperative Recovery in Children (PRiC) questionnaire. Caregivers assessed their child's pain, anxiety, and nausea on a numeric analog scale and kept a log of analgesic administration.

**Results:**

High pain levels (FPS-R ≥ 4) were reported in all surgical and age groups (TT ± A age 4–11, TE ± A age 4–11, TE ± A age 12–17), but there were variations in pain intensity and duration within and between groups. The TE ± A group scored more days with moderate to very excruciating pain and lower recovery than the TT ± A group, with the worst outcomes reported by older TE ± A children. The majority of the children used paracetamol + COX-inhibitors at home, but regular administration of analgesics was lacking, particularly during late evening and at night. Few were received rescue medication (opioid or clonidine) despite severe pain. Physical symptoms and daily life activities were affected during the recovery period. There was moderate agreement between child and the caregiver’s pain assessment scores.

**Conclusion:**

Children reported a troublesome recovery with significant postoperative pain, particularly older children undergoing tonsillectomy. Pain treatment at home was suboptimal and lacked regular analgesic administration. Patient information needs to be improved regarding the importance of regular administration of analgesics and rescue medication.

## Introduction

The recovery period following pediatric tonsil surgery is long, approximately 14 days, but the time spent in the hospital is short [1]. Thus, recovery mainly takes place in a home setting, affecting daily life activities, physical symptoms, and emotional aspects [2]. Recovery from tonsil surgery involves a large amount of pain and appears to be more troublesome than other types of childhood surgical procedures [3, 4]. In addition to the suffering associated with pain, high levels of postsurgical pain increase the risk of developing short- and long-term complications, such as delayed behavioral and clinical recovery, including persistent long-term pain [3, 5].

Exposure to high levels of pain and vulnerability in the pediatric population makes pain management essential during recovery from tonsil surgery. Well-established principles for successful management of postoperative pain include multimodal analgesia, adequate dosage, administration at regular intervals, use of appropriate route of administration [1, 4, 6], and complementary methods include different distraction techniques and consuming cold food or drink. Implementing these principles in home care places significant demands on the caregivers and the child, as they necessitate accurate assessment and correct analgesic dosing.

Tonsil surgery is repeatedly identified as a procedure with a high incidence of prolonged postsurgical pain with suboptimal administration of analgesics at home [1, 7]. However, over the last decade, improvement efforts have been made by the implementation of guidelines and recommendations [8, 9]. Despite clinical care guidelines, many children continue to experience unnecessary postoperative pain [7]. Data from the National Tonsil Surgery Registry in Sweden shows a high frequency of unplanned health care contacts due to undertreated pain [10]. The pain outcome may depend on the families' compliance with the administration of analgesics. To further optimize the care of these children, increased knowledge regarding postoperative pain and analgesic administration at home is necessary. In the evaluation, self-report of pain is considered the gold standard, but parents have a central role in managing the child´s pain at home, and caregivers’ assessment of the child's pain together with the child's assessment is of great importance. The aim of this study was to explore the severity and duration of postoperative pain, the management of analgesics and postoperative recovery in children undergoing tonsil surgery.

## Materials and methods

This prospective cohort study was approved by the Research Ethics Committee of Uppsala (2017/169).

### Participants and procedure

The data collection was performed from November 2017 to November 2019. A consecutive sample of children aged 4–17 years scheduled to undergo elective tonsillectomy or tonsillotomy (with or without adenoidectomy) was invited to participate. The exclusion criteria were the child and/or caregivers not able to speak and/or understand Swedish and the child having psychiatric or developmental disorders or physical conditions interfering with the standard care program. The participants were recruited from six ear–nose–throat (ENT) clinics, four hospitals and two private day-surgery clinics, in Sweden (Table 1). Guided by a previous study [11], the hospitals were chosen based on their instructions for postoperative pain management at home after tonsil surgery. The purpose was to obtain a sample reflecting the most current pain treatment regimen in Sweden.

**Table 1:** Short description of pain medication instructions provided by each participating hospital site for caregivers to manage their child´s postoperative pain

Hospital (*n*^a^)	Instructions
1 (*n* = 120)	Younger children (< 50 kg): Administer paracetamol and ibuprofen according to dosages recommended in the Swedish national guidelines.^b^ (The information was given verbally and in writing). Older children (> 50 kg): Administer paracetamol and naproxen as recommended on the label packaging. Caregivers may administer morphine to older children who have undergone tonsillectomy. (The information was given verbally and in writing)
2 (*n* = 26)	All children: Administer paracetamol and ibuprofen as recommended on the label packaging. (The information was given verbally)
3 (*n* = 98)	All children: Administer paracetamol and ibuprofen as recommended on the label packaging. Instruction to alternate between paracetamol and ibuprofen (every third hour). (The information was given verbally and in writing)
4 (*n* = 128)	All children: Administer paracetamol and ibuprofen according to the dosages recommended in the Swedish national guidelines.^b^ Caregivers may administer clonidine to children who have undergone tonsillectomy. (The information was given verbally and in writing)
5 (*n* = 141)	Younger children (< 50 kg): Administer paracetamol and ibuprofen as recommended on the label packaging (The information to the younger children was given verbally). Older children (> 50 kg): Administer paracetamol and ibuprofen as recommended on the label packaging. Caregivers may administer clonidine to older children who have undergone tonsillectomy. (The information to older children was given verbally and in writing)
6 (*n* = 218)	All children: Administer paracetamol and ibuprofen according to the dosages recommended in the Swedish national guidelines.^b^ Caregivers may administer clonidine to children who have undergone tonsillectomy. (The information was given verbally and in writing)

^a^*n* = number of tonsil surgeries performed in children aged 4–17 years in 2018, data from the National Tonsil Surgery Registry in Sweden

^b^A description of the Swedish national guidelines is available in Ericsson et al. 2015^16^

The children were invited to participate together with their caregivers, and they were informed of the study through an information letter that was sent with the notification about the operation. Informed consent was obtained from interested caregivers and children on the day of surgery. Following consent, a pain diary with a prepaid envelope was handed to the families. The families were asked to fill in the diary until the child stopped using analgesics and was completely free of pain or for a maximum of 12 days. A text message reminder was sent 1–2 days after the surgery to remind them to complete and return the diary.

The anesthetic and surgical procedure followed the standard care at each of the six hospitals. After the surgery, the recovery care unit nurse or the surgeon explained the discharge instructions and provided directions for at-home postoperative pain management to the caregivers and the child. Pain management varied among the six hospitals (Table 1). Surgical methods, anesthesia procedures, and instructions for pain treatment at home were collected from the medical records.

### Measures

Data were collected through a diary to be completed by both the child and the caregiver(s). The child and the parent sections of the diary were completed separately. The layout of the child section was playful with cartoon characters. Each postoperative day had its own book spread containing instruments and measurements for the previous 24 h. The diary had a cover page with instructions and explanations for how to explain the instruments to the child. The caregivers were encouraged to help the child complete their section of the diary, emphasizing the child’s assessment to be recorded.

#### Child-reported measures—pain intensity and postoperative recovery

The children reported pain intensity five times a day using the Faces Pain Scale-Revised (FPS-R), a rating scale using six gender-neutral pictorial facial representations of increasing pain severity. The FPS‐R has been shown to be a reliable and valid measure of pain intensity in children aged 4–17 years [12].

Postoperative recovery was reported once a day (in the evenings) by the children using the Postoperative Recovery in Children (PRiC) questionnaire [13]. The PRiC assesses 23 aspects of recovery including, daily life activities, physical symptoms and emotional aspects. The items concern the previous 24 h and are assessed on a four-grade scale (1 = ”not at all”, 2 = “little”, 3 = ”much”, and 4 = “very much”). The PRiC also includes one item of a more general nature addressing the child’s current general health, which is answered by 1 = ”very well”, 2 = “pretty well”, 3 = ”pretty bad”, or 4 = ”very bad”. The PRiC contains photo illustrations linked to each item, which were optional. The PRiC was developed and tested in a Swedish context of children undergoing tonsil surgery and is a valid and reliable questionnaire [2].

#### Parent-reported measures—pain intensity, anxiety, nausea, and daytime tiredness

The caregivers registered the child’s postoperative pain intensity, anxiety and nausea five times a day on a 10-point numeric rating scale (NRS) ranging from 0 = “no pain” (or anxiety or nausea) to 10 = “worst possible pain” (or anxiety or nausea). For pain, the NRS can be considered functionally equivalent to the FPS-R [14].

Furthermore, the caregivers assessed the child’s daytime tiredness five times a day by answering the question, “I experience that my child is more tired than usual (compare with how tired your child was at this time of day before surgery)”. The question was answered using a 4-point Likert scale (1 = “not at all”, 2 = “little, 3 = “much”, and 4 = “very much”). The caregivers were also asked to record the date, time, medication type, and dose for each analgesic administration.

### Study variables

The children were divided by surgical method and age: tonsillotomy with or without adenoidectomy in those aged 4–11 years (TT ± A age 4–11), tonsillectomy with or without adenoidectomy in those aged 4–11 years (TE ± A age 4–11), and tonsillectomy with or without adenoidectomy in those aged 12–17 years (TE ± A age 12–17). Older children (age 12–17) undergoing TT ± A were few (*n* = 5) and are presented only in the context of the total cohort and not analyzed as a separate group. The regularity of analgesic administration, agreement between the caregivers’ and the children’s pain scores, parent-scored anxiety, nausea and day fatigue were analyzed for days 1–3, and the PRiC items are from day 3. Days 1–3 were chosen to illustrate the most pain-intensive period. In this study, pain intensity was categorized as follows: FPS-R/NRS = 0 indicates “no pain”, FPS-R/NRS = 1–3 indicates “mild pain”, FPS-R/NRS = 4–7 indicates “moderate” to “severe” pain, and FPS-R/NRS = 8–10 indicates “very severe” to “excruciating pain”. Day 1 refers to the day after surgery.

### Statistical analyses

Continuous variables are described by the mean, standard deviation (SD), median, minimum and maximum and categorical data by numbers and percentages. For comparisons between two groups, *t* tests were used for continuous variables, Mantel–Haenszel Chi-square tests were used for ordered categorical variables, and Fisher’s exact tests were used for dichotomous data. The mean differences with 95% confidence intervals (CI) were assessed with *t* tests. In line with previous research [15, 16], the agreement between the parent daily median NRS scores and their child’s daily median scores on the FPS-R was set to < 2 points difference. Furthermore, a weighted kappa (K_w_) of all child–parent pain scores during days 1–3 was calculated. The correlations between parent-reported pain intensity and anxiety, pain intensity and nausea, and pain intensity and fatigue were analyzed with Spearman’s rank correlations (r_s_). The statistical analyses were performed using IBM SPSS Statistics version 25. All significance tests were two-sided and conducted at the 5% significance level.

## Results

A total of 299 children and caregivers returned the diaries and were included in the analyses (response rate: 58.2%). Compared to those who did not return the diary, the children who returned the diary did not differ in age, sex or surgery method but differed in indication for surgery (obstruction: 78% vs. 69%, *p* = 0.025). The children’s ages, sex, and surgical indication in the total cohort and each surgical and age group are presented in Table 2.

**Table 2:** Demographic data, surgical indication, pain score and duration of postoperative analgesic administration for the total cohort and each surgical method, and age groups

	Total *n* = 299^a^	TT ± A age 4–11 *n* = 168	TE ± A age 4–11 *n* = 78	TE ± A age 12–17 *n* = 48	TT ± A age 4–11 *vs. *TE ± A age 4–11 mean difference (CI) *p* value	TE ± A age 4–11 *vs.* TE ± A age 12–17 mean difference (CI) *p* value	TT ± A age 4–11 *vs.* TE ± A age 12–17 mean difference (CI) *p* value
**Sex**							
Male *n* (%)	150 (50.2%)	84 (50.0%)	41 (52.6%)	22 (45.8%)			
Female *n* (%)	149 (49.8%)	84 (50.0%)	37 (47.4%)	26 (54.2%)	–(−) 0 .784	– (−) 00.467	– (−) 0.627
**Age**							
Mean (SD)	7.7 (3.8)	5.8 (1.8)	7.1 (2.2)	14.8 (1.9)	– (−) < 0.001	– (−) < 0.001	– (−) < 0.001
Median (q1:q3)	6 (5:10)	5 (4:7)	6 (5:9)	15 (12:17)			
Min/max	4/17	4/11	4/11	12/17			
**Main indication**							
Infectious causes^c^	66 (22.1%)	0 (0%)	31 (39.7%)	34 (70.8%)			
Obstruction	233 (77.9%)	168 (100%)	47 (60.3%)	14 (29.2%)	– (−). < 0.001	– (−) 0.001	– (−) < 0.001
**Number of days with FPS-R score ≥ 4**^b^							
Mean days (SD)	4.5 (3.3)	2.9 (2.4)	6.2 (3.0)	7.9 (2.4)	3.3 (2.6;4.0) –	1.7 (0.7;2.7) –	5.0 (4.2;5.8) –
0–2 days *n* (%)	97 (33.2%)	88 (53.0%)	6 (7.8%)	2 (4.5%)			
3–5 days *n* (%)	83 (28.4%)	52 (31.3%)	28 (36.4%)	1 (2.3%)			
6–8 days *n* (%)	69 (23.6%)	21 (12.7%)	23 (29.9%)	23 (52.3%)			
> 8 days *n* (%)	43 (14.7%)	5 (3.0%)	20 (26.0%)	18 (40.9%)	– (−) < 0.001	– (−) 0.001	– (−) < 0.001
Missing value *n*	7	2	1	4			
**Number of days with FPS-R score ≥ 8**^b^							
Mean (SD)	1.6 (2.2)	0.8 (1.4)	2.4 (2.5)	3.3 (2.7)	1.6 (0.9;2.2) –	0.9 (− 0.1;1.9) –	2.4 (1.6;3.3) –
0–2 days *n* (%)	228 (78.1%)	150 (90.4%)	52 (67.5%)	22 (50.0%)			
3–5 days *n* (%)	42 (14.4%)	11 (6.6%)	17 (22.1%)	14 (31.8%)			
6–8 days *n* (%)	16 (5.5%)	4 (2.4%)	5 (6.5%)	6 (13.6%)			
> 8 days *n* (%)	6 (2.1%)	1 (0.6%)	3 (3.9%)	2 (4.5%)	– (−) < 0.001	– (−) 0.096	– (−) < 0.001
Missing value *n*	7	2	1	4			
**Number of days with analgesics**							
Mean (SD)	7.1 (3.0)	5.7 (2.3)	8.4 (2.8)	10.2 (2.3)	2.6 (1.9;3.4) –	1.8 (0.9;2.8) –	4.5 (3.6;5.2) –
0–2 days *n* (%)	9 (3.2%)	7 (4.3%)	2 (2.8%)	0 (0%)			
3–5 days *n* (%)	93 (33.1%)	79 (48.5%)	11 (15.3%)	2 (4.9%)			
6–8 days *n* (%)	86 (30.6%)	61 (37.4%)	20 (27.8%)	5 (12.2%)			
> 8 days *n* (%)	93 (33.1%)	16 (9.8%)	39 (54.2%)	34 (82.9%)	– (−) < 0.001	– (−) 0.003	– (−) < 0.001
Missing value *n*	18	5	6	7			

*T* test on continuous data (Age), Fisher’s exact test on dichotomous data (sex and indication). Mantel Haenzsel Chi-square test for ordered categorical data (number of days with FPS-R score ≥ 4, FPS-R score ≥ 8 and days with analgesics). 95% CI for mean differences were based on T test

*TE ± A* tonsillectomy with or without adenoidectomy, *TT ± A* tonsillotomy with or without adenoidectomy, *SD* standard deviation, *q1* quartile 1, *q3* quartile 3, *FPS-R* face pain scale-revised

^a^Older children (age 12–17) undergoing TT ± A were few (*n* = 5) and are presented only in the context of the total cohort and not analyzed as a separate group

^b^FPS-R ≥ 4 or FPS-R ≥ 8 in at least one daily assessment

^c^Recurrent tonsillitis, chronic tonsillitis

### Severity and duration of postoperative pain

**Fig. 1:**
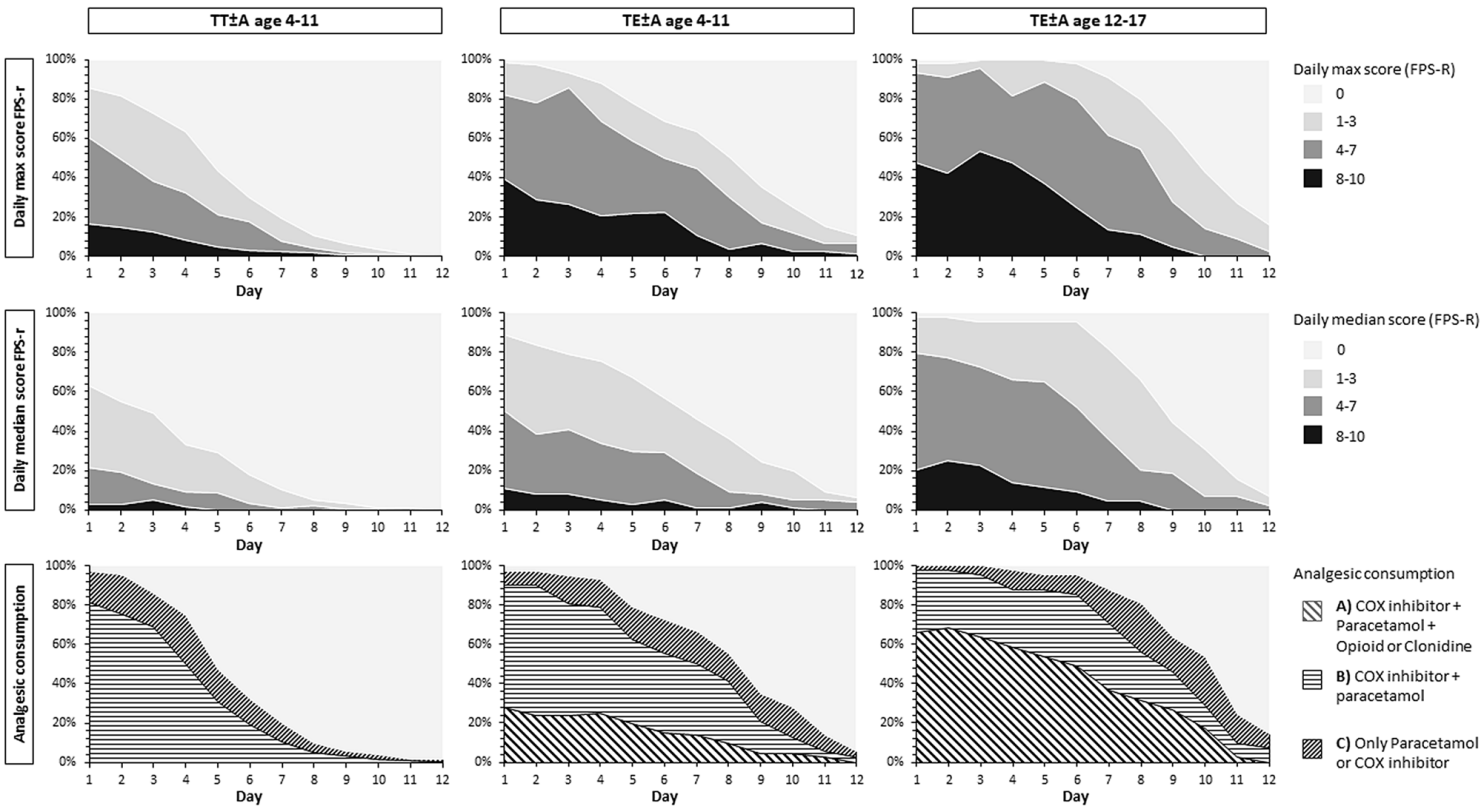
The top row illustrates the percentage of children reporting a daily pain max score 0, 1–3, 4–7 or 8–10 with Face Pain Scale-Revised (FPS-R) day 1–12 after surgery. The middle row illustrates the percentage of children reporting a daily pain median score 0, 1–3, 4–7 or 8–10 with FPS-R day 1–12 after surgery. The bottom row illustrates the percentage of children who used, paracetamol + COX inhibitor + opioid or clonidine, paracetamol + COX inhibitor, only paracetamol or COX inhibitor, day 1–12

The children in all surgical and age groups reported events of moderate to excruciating pain (FPS-R ≥ 4) (Fig. 1). The greatest number of days with FPS-R ≥ 4 (in at least one daily assessment) was reported by children in the TE ± A age 12–17 group (mean days: 7.9, SD: 2.4), followed by the TE ± A age 4–11 group (mean days: 6.2, SD: 3.0), while the fewest number of days with FPS-R ≥ 4 was reported by children in the TT ± A age 4–11 group (mean days: 2.9, SD: 2.4).

There was also a significant difference in the number of days with pain FPS-R ≥ 8 (in at least one daily assessment) between the TT ± A and TE ± A groups (Table 2). No sex differences in the number of days with FPS-R ≥ 4 or the number of days with FPS-R ≥ 8 in the respective surgical and age groups. Additionally, no significant differences in the number of days with FPS-R ≥ 4 or the number of days with FPS-R ≥ 8 were observed between children undergoing TE ± A due to obstruction and children undergoing TE ± A for infectious causes (e.g., recurrent tonsillitis, and chronic tonsillitis).

**Fig. 2:**
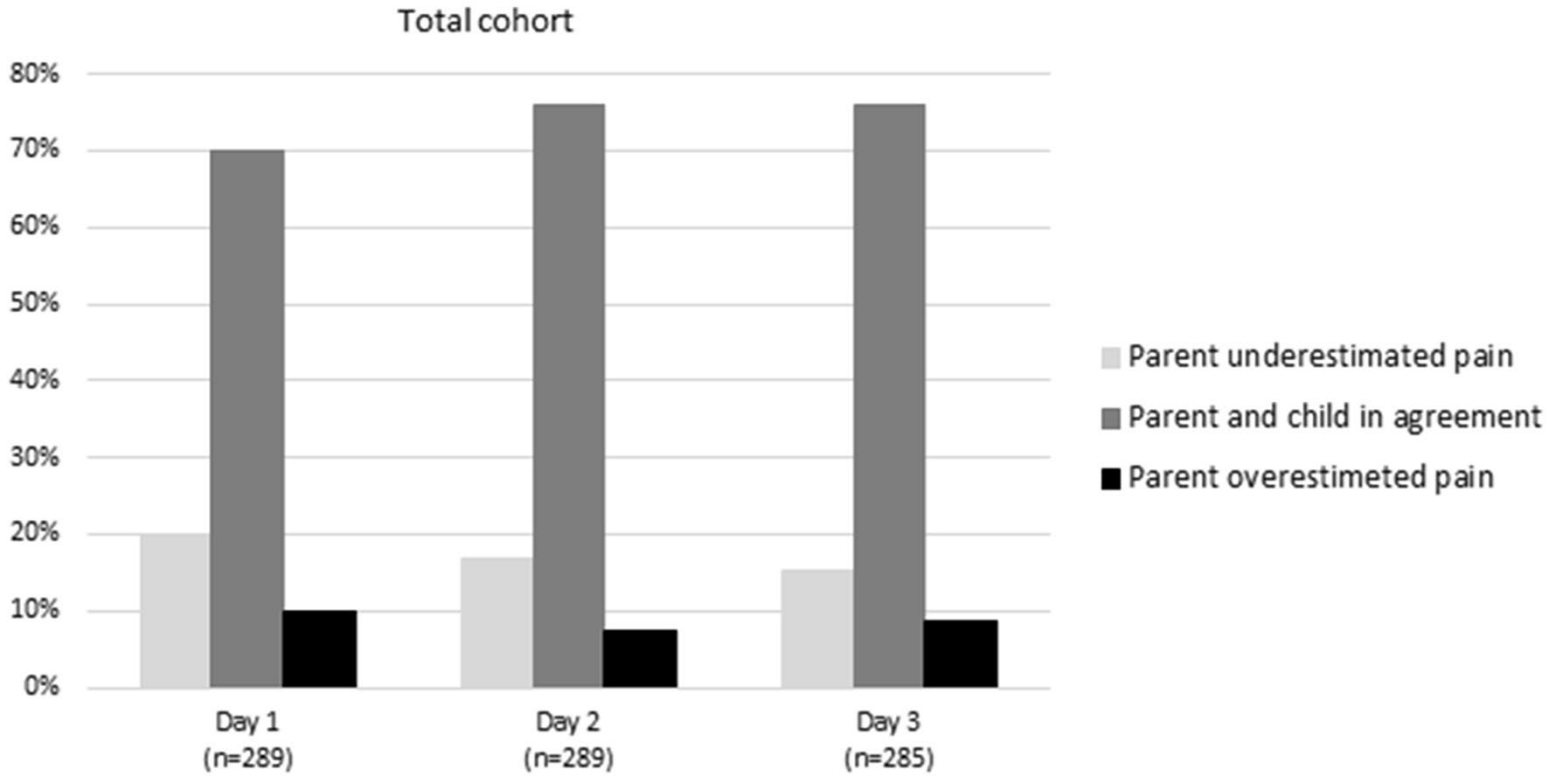
The percentage of child–parent agreement and disagreement when comparing each child’s daily median score on FPS-R with their parent’s median score on NRS. The agreement was defined as < 2 point difference between the median score

There was a moderate agreement between the child’s and the caregivers´ pain assessment. When comparing each child´s daily median score on the FPS-R with their caregivers’ daily median score on the NRS, the majority of the child–parent pairs (69–74%) agreed (< 2 point difference between NRS/FPS-R scale scores) on days 1–3 after surgery. Approximately, 16–21% of the caregivers underestimated and approximately 9–11% overestimated (≥ 2-point NRS/FPS-R difference) their child’s pain on days 1–3 after surgery (Fig. 2). There was no significant difference in the percentage of child–parent disagreement between the older and younger children (ages 12–17 vs. 4–11). A weighted kappa analysis of all child–parent pain assessments during days 1–3 resulted in a kappa coefficient of 0.57.

### Analgesic management

The majority of the children in each surgical and age group followed a multimodal pain treatment regimen (Fig. 1). The TE ± A age 12–17 group reported the longest period of postoperative analgesic administration (mean: 10.2 days, SD: 2.3), followed by the TE ± A age 4–11 group (mean: 8.4 days, SD: 2.8), while the shortest period of analgesic use was reported in the TT ± A age 4–11 group (mean: 5.7 days, SD: 2.3) (Table 2). The majority (92%, *n* = 60/65) of the children who had access to clonidine or opioids used them for several days (mean days with opioid: 6.9, SD 3.0; mean days with clonidine: 5.4, SD 2.7).

Regular analgesic administration was lacking, particularly during late evening and at night. Among paracetamol users, approximately half of the children received paracetamol doses four times per 24 h period during days 1–3 after surgery (day 1: 52%, day 2: 45%, day 3: 42%). Among ibuprofen users, approximately one-third of the children were administered ibuprofen doses four times a day (i.e., per 24 h) during days 1–3 after surgery (day 1: 34%, day 2: 31%, day 3: 29%). Between 9 pm on day 1 and 8 am on day 2 after surgery, 24% of the children in the total cohort were not administered any analgesics. There was a similar proportion (27%) of non-users of analgesics between day 2 (9 pm) and day 3 (8 am).

There was a lack of adherence to the prescribed daily dose of paracetamol; the most common discrepancy was undertreatment. The difference in the percentage between the prescribed and administered daily (24 h) dose of paracetamol was a mean of -26 percentage points on day 1, -31percentage points on day 2, and -38 percentage points on day 3. There were two children who were administered a daily (24 h) dose of paracetamol > 100 mg/kg on day 1 after surgery (child 1: 120 mg/kg, child 2: 108 mg/kg).

### Postoperative recovery

According to the PRiC self-reports, there were higher scores, i.e., worse recovery, in the TE group. To examine clinically significant differences, the proportion of children who reported “much” or “very much” for particular PRiC items on day three after surgery are tabulated in Table 3, and the subgroup analysis is shown in Table 4. In the total cohort, the items with the highest proportion of “much” or “very much” answers were sore throat, difficulties eating, difficulties playing/being active, earache, and difficulties talking. For each of these five items, the TE ± A age 12–17 group had the highest proportion of “much” or “very much”, while the lowest proportion was reported by TT ± A age 4–11 group (Table 3). Among the top five items, sex differences were noted in the TE ± A age 4–11 group; the boys had a higher proportion of “much” or “very much” than girls for difficulties eating (41% vs. 14%, *p* = 0.010) and sore throat (66% vs. 38%, *p* = 0.021). In both age groups, there was no significant difference in PRiC items between the children undergoing TE ± A due to obstruction and those undergoing TE ± A for infectious causes (Table 4).

**Table 3:** Day three after surgery, percentage (%) of the children answering that they within the previous 24 h experienced/had difficulties: “much “or “very much”, or “pretty bad” or “very bad” (*last item*), in the PRiC- items

		Third day (24 h)				
	TT ± A age 4–11 *n* = 168	TE ± A age 4–11 *n* = 78	TE ± A age 12–17 *n* = 48	*p* value^a^ TE ± A age 4–11 * vs.* TT ± A age 4–11	*p* value^a^ TE ± A age 12–17 *vs.* TE ± A age 4–11	*p* value^a^ TE ± A age 12–17 *vs.* TT ± A age 4–11
Sore throat	20.9% (33/158)	52.0% (39/75)	77.1% (37/48)	< 0.001	0.007	< 0.001
Eating	8.9% (14/158)	28.8% (21/73)	50.0% (24/48)	< 0.001	0.022	< 0.001
Playing/being active	8.1% (13/160)	24.0% (18/75)	52.1% (25/48)	0.002	0.002	< 0.001
Earache	5.0% (8/160)	18.7% (14/74)	33.3% (16/48)	0.001	0.087	< 0.001
Talking	1.9% (3/159)	12.0% (9/75)	41.7% (20/48)	0.002	< 0.001	< 0.001
Sadness	8.2% (13/159)	6.7% (5/75)	4.2% (2/48)	0.78	0.704	0.53
Stomach ache	3.8% (6/159)	10.8% (8/74)	6.3% (3/48)	0.071	0.52	0.44
Brushing teeth	2.5% (4/157)	8.5% (6/71)	10.9% (5/46)	0.074	0.75	0.030
Nausea	1.9% (3/158)	8.0% (6/75)	10.4% (5/48)	0.033	0.75	0.018
Defecations	1.9% (3/155)	2.7% (2/73)	17.0% (8/47)	0.66	0.013	< 0.001
Headache	5.0% (8/159)	2.7% (2/74)	4.2% (2/48)	0.51	0.65	1
Dizziness	0.0% (0/158)	2.7% (2/74)	18.8% (9/48)	0.10	0.007	< 0.001
Resting	4.5% (7/157)	2.7% (2/74)	4.2% (2/48)	0.72	0.65	1
Feeling cold	2.5% (4/157)	5.3% (4/75)	4.2% (2/48)	0.28	1	0.63
Sleeping	1.9% (3/160)	1.4% (1/74)	4.2% (2/48)	1	0.56	0.33
Breathing	1.3% (2/159)	0.0% (0/74)	6.3% (3/48)	1	0.059	0.083
Frightening dreams	2.5% (4/159)	1.4% (1/74)	0.0% (0/48)	1	1	0.58
Washing/showering	0.0% (0/157)	2.9% (2/68)	2.2% (1/45)	0.090	1	0.22
Vomiting	0.0% (0/158)	1.3% (1/75)	2.1% (1/48)	0.32	1	0.23
Urination	0.0% (0/159)	0.0% (0/74)	2.1% (1/48)	–	0.39	0.23
Blood in mouth	0.0% (0/160)	0.0% (0/74)	0.0% (0/48)	–	–	–
At the moment I feel	12.9% (19/147)	22.9% (16/70)	35.6% (16/45)	0.076	0.20	0.002

The denominator is the number of answers to the question

*TE ± A* tonsillectomy with or without adenoidectomy, *TT ± A* tonsillotomy with or without adenoidectomy, *FPS-R* face pain scale-revised

^a^Fisher’s exact test

**Table 4:** Day three after surgery, percentage (%) of the children answering that they within the previous 24 h experienced/had difficulties: “much “or “very much”, in the top five PRiC items (guided by Table 3): subgroup analysis by sex, surgical indication and pain score

	Third day (24 h)								
	TT ± A age 4–11			TE ± A age A 4–11			TE ± A age 12–17		
Sex	Boys	Girls	*p* value^a^	Boys	Girls	*p* value^a^	Boys	Girls	*p* value^a^
Sore throat	19.5% (15/77)	22.2% (18/81)	0.70	65.8% (25/38)	36.8% (14/38)	0.021	81.2% (18/22)	73.1% (19/26)	0.514
Eating	7.7% (6/78)	10.0% (8/80)	0.78	42.1% (16/38)	13.9% (5/36)	0.010	50.0% (11/22)	50.0% (13/26)	1
Playing/being active	7.6% (6/79)	8.6% (7/81)	1	31.6% (12/38)	15.8% (6/38)	0.176	63.6% (14/22)	42.3% (11/26)	0.16
Earache	2.5% (2/79)	7.4% (6/81)	0.28	13.2% (5/38)	24.3% (9/37)	0.249	31.8% (7/22)	34.6% (9/26)	1
Talking	2.6% (2/78)	1.2% (1/81)	0.62	10.5% (4/38)	13.2% (5/38)	1	45.5% (10/22)	38.5% (10/26)	0.770

Surgical indication	Obstruction	Infection	*p* value^a^	Obstruction	Infection	*p* value^a^	Obstruction	Infection	*p* value^a^
Sore throat	20.9% (33/158)	–	–	53.2% (25/47)	48.3% (14/29)	0.81	71.4% (10/14)	79.4% (27/34)	0.71
Eating	8.9% (14/158)	–	–	22.2% (10/45)	37.9% (11/29)	0.19	57.1% (8/14)	47.1% (16/34)	0.75
Playing/being active	8.1% (13/160)	–	–	23.4% (11/47)	24.1% (7/29	1	64.3% (9/14)	47.1% (16/34)	0.35
Earache	5.0% (8/160)	–	–	19.6% (9/46)	17.2% (5/29)	1	28.6% (4/14)	35.3% (12/34)	0.75
Talking	1.9% (3/159)	–	–	12.8% (6/47)	10.3% (3/29)	1	50.0% (7/14)	38.2% (13/34)	0.53

Pain score (Median^b^)	FPS-R < 4	FPS-R ≥ 4	*p* value^a^	FPS-R < 4	FPS-R ≥ 4	*p* value^a^	FPS-R < 4	FPS-R ≥ 4	*p* value^a^
Sore throat	10.4% (14/135)	82.6% (19/23)	< 0.001	29.5% (13/44)	80.6% (25/31)	< 0.001	41.7% (5/12)	90.9% (30/33)	0.001
Eating	3.7% (5/163)	40.9% (9/22)	< 0.001	13.6% (6/44)	48.3% (14/29)	0.003	16.7% (2/12)	63.6% (21/33)	< 0.001
Playing/being active	5.1% (7/137)	26.1% (6/23)	0.004	11.4% (5/44)	41.9% (13/31)	0.005	33.3% (4/12)	57.6% (19/33)	0.189
Earache	3.6% (5/137)	13.0% (3/23)	0.090	6.8% (3/44)	36.7% (11/30)	0.002	33.3% (4/12)	33.3% (11/33)	1
Talking	0.0% (0/136)	13.0% (3/23)	0.003	4.5% (2/44)	22.6% (7/31)	0.028	8.3% (1/12)	51.5% (17/33)	0s.014

The denominator is the number of answers to the question

*TE ± A* tonsillectomy with or without adenoidectomy, *TT ± A* tonsillotomy with or without adenoidectomy, *FPS-R* face pain scale-revised

^a^Fisher’s exact test

^b^Median score on FPS-R day three after surgery

The correlation between parent reports of the child´s pain intensity and anxiety on days 1–3 was r_s_ = 0.34–0.53, between pain intensity and daytime tiredness on days 1–3 was r_s_ = 0.45–0.59 and between pain intensity and nausea on days 1–3 was r_s_ = 0.21–0.35.

Nausea was mainly reported on the day of surgery. On the day of surgery, the caregivers reported nausea ≥ 4 (in at least one daily assessment) for 23% in the TT ± A age 4–11 group, 34% in the TE ± A age 4–11 group and 19% in the TE ± A age 12–17 group. The first day after surgery, the reported nausea ≥ 4 (in at least one daily assessment) was reduced to 2% in the TT ± A age 4–11 group, to 8% in the TE ± A age 4–11 group and to 10% in the TE ± A age 12–17 group.

## Discussion

Children and caregivers report a painful recovery after tonsil surgery that affects daily life. Despite significant pain scores and troublesome recovery, the management of analgesics is often suboptimal and unsatisfactory.

The high levels of pain and early discharge from the hospital necessitate effective pain management at home. Inadequately managed postoperative pain at home affects both physiological and psychological functions, such as poor oral intake, sleep disturbances, anxiety, and behavioral changes [3]. Issues also recognized in our study with children’s PRiC-score and caregivers report (NRS-score) of their child’s anxiety.

High pain levels for several days were reported by all groups and foremost in children with tonsillectomy, demonstrating the need for more effective pain management to reach acceptable pain levels and improve recovery. However, pain intensity, pain duration, and scores on several PRiC items differed between the surgical groups, which can be related to the amount of tissue damage. In tonsillectomy, the entire tonsil and its capsule is removed, while in tonsillotomy, only the medial portions of the tonsils are removed, leaving the tonsil capsule intact [17]. Older children reported higher pain levels after tonsillectomy than younger children in line with previous studies [18], but the results should be interpreted with caution because children’s perception of pain and the way they behave when in pain is influenced by age and cognitive development [19]. Regarding pain levels, there is a need to clarify that pain is a subjective experience, and an acceptable pain level after tonsil surgery is a matter of the individual child. With proper pain management, there is no need for children to be experiencing events of moderate to severe pain. Reduction in pain enables improved recovery.

The six ENT clinics in the present study used somewhat different recommendations for pain management at home. All children were instructed to take paracetamol and COX inhibitor. However, the dosage of paracetamol, type of COX inhibitor, and use of rescue medication (opioids/clonidine) differed among the ENT clinics. Professionals may give adequate and evidence-based instructions, but the full potential of treatment does not necessarily reach the children. The present study showed that the frequency of analgesic dosing was not adequate throughout the 24-h day, particularly during late evening and at night, resulting in low adherence to the recommended daily dose. The effect of low compliance could explain the less effective pain outcomes. There is a consensus that analgesics should be preemptively and regularly administered to reduce pain and that early effective management is more effective than treating breakthrough pain [1, 6]. However, there is likely self-regulation that less pain in the patient leads to the administration of less analgesics.

Pain scores after tonsil surgery remain high during the first postoperative days, and analgesics in addition to paracetamol and COX inhibitor seem to be needed. This is particularly important in the treatment of older children undergoing tonsillectomy, where almost half of the children scored severe to excruciating pain for several days. However, it is important to note that there were also younger children who had undergone tonsillotomy and reported unacceptably high pain levels. Most children who had access to rescue medication (opioids/clonidine) used them repeatedly for several days. After the implementation of national guidelines in Sweden [9], the use of clonidine has become more common, and they are used more often than opioids as rescue medication following tonsil surgery [11]. In our clinical practice, clonidine is an effective analgesic as part of the management of postoperative pain and has fewer side effects than opioids. The most serious side effect of opioids is respiratory depression, and fatal outcome has been described when used after tonsil surgery for pain relief [20].

Several barriers to effective pain management after discharge have been previously identified including both inadequate administration and inadequate prescription. Inadequate administration involves aspects of the caregiver, for example, the ability to assess and recognize pain and having misconceptions about analgesics. However, there are also child-related factors, such as refusing to take the medication. Inadequate prescription includes inadequate dosing, choice of analgesics and appropriate patient formulations, as well as system factors such as poor discharge instructions and difficulty obtaining medication [1]. Some of these factors are described in the present study, such as caregivers' ability to recognize/assess pain and poor discharge instructions. In the present study, caregivers' ability to recognize and assess their child's pain was moderate (K_W_ = 0.57) [21], and the most common disagreement was caregivers underestimating the pain. On the other hand, overestimating pain by caregivers might cause problems with the use of high doses of analgesics [15]. The most serious risk is opioid overdosing. These safety aspects are important and are included in the Swedish national guidelines [9].

Some ENT clinics in this study had poor and unclear discharge analgesic instructions. To address caregivers’ misconceptions about medications and children's unwillingness to take analgesics, the information needs to at least include the effects of the analgesics, importance of regular administration, side effects, and alternative routes of administration. The efficacy and feasibility of around-the-clock (ATC) dosing for postoperative pain management at home have been investigated in several studies [1, 8] and can be one of several interventions to optimize pain management. An approach to increase compliance could be to supply a package of analgesics, e.g., prepared by a pharmacist, for basic daily treatment and rescue medication and not only giving prescriptions.

One of the most important morbidities associated with tonsil surgery is postoperative nausea and vomiting (PONV) [8]. To improve recovery, PONV should be taken into account. This study showed that nausea and vomiting were most prominent during the initial postoperative phase. Nausea can be limited by the choice of anesthesia performed, minimizing blood running down to the ventricle and the use of PONV prophylaxis. Corticosteroids, betamethasone, is regularly used as PONV prophylaxis in Sweden and was part of the anesthetic management in the clinics taking part in the present study.

The strengths of the current study were the large number of participants from six ENT clinics and a long follow-up. The study detailed the pain situation at home after tonsil surgery based on reports from both the children and the parents. The limitations included the low response rate and no knowledge of the recovery process among the non-responders. It can be assumed that families who had the organizational strength to complete and return the diary also had better adherence to analgesic treatment compared to families who did not return the diary. However, some results (duration of analgesics) were similar to randomized controlled trials (RCTs) and register studies [10, 17]. The present study did not include highly sensitive measures to identify the impact of different analgesic regimens (e.g., clonidine), which is why there is still no consensus as to the most effective postoperative pain control regimen after tonsil surgery. Future studies are needed regarding rescue medication for optimal pain management with a minimum of side effects. Neither did the study take into account complementary nonpharmacological interventions or behavioral approaches, including relaxation and distraction, during the postoperative period. More qualitative studies are required to provide insight into the caregivers’ and children’s experiences and management of the child’s postoperative pain at home.

## Conclusion

Children report (in moderate accordance with caregiver assessments) a troublesome recovery with significant postoperative pain, particularly older children undergoing tonsillectomy. The present study suggests that pain control after tonsil surgery could be improved. The full potential of treatment is not realized due to an insufficient dosage frequency and underuse of rescue medication. Patient information needs to be improved regarding the importance of regular administration of analgesics and rescue analgesics. For most of the younger children, multimodal analgesia with paracetamol and COX inhibitor seems to be adequate after tonsil surgery. For an older age group, a combination of paracetamol and COX inhibitor is not satisfactory enough in managing postoperative pain. We suggest preferably to add clonidine as part of the basic analgesic medication given around the clock in patients undergoing tonsillectomy. Rescue medication, with high safety and limited side effects, should always be prescribed to manage breakthrough pain.
